# Metal Binding Properties of the N-Terminus of the Functional Amyloid Orb2

**DOI:** 10.3390/biom7030057

**Published:** 2017-08-01

**Authors:** Thalia H. Bajakian, Silvia A. Cervantes, Maria A. Soria, Maïwenn Beaugrand, Ji Yun Kim, Rachel J. Service, Ansgar B. Siemer

**Affiliations:** 1 Department of Biochemistry & Molecular Medicine and the Zilkha Neurogenetic Institute, Keck School of Medicine of USC, Los Angeles, CA 90033, USA; t.bajakian@gmail.com (T.H.B.); angie.crvts@gmail.com (S.A.C.); mariaaco@usc.edu (M.A.S.); maiwenn.beaugrand@gmail.com (M.B.); kim821@usc.edu (J.Y.K.); servicerj@gmail.com (R.J.S.); 2 Department of Biomedical Engineering, McGill University, Montreal, QC H3A 2B4, Canada; 3 U.S. Army Construction Engineering Research Laboratory, 2902 Newmark Dr, Champaign, IL 61822, USA

**Keywords:** amyloid, protein–metal interaction, aggregation, ITC, thioflavin T fluorescence

## Abstract

The cytoplasmic polyadenylation element binding protein (CPEB) homologue Orb2 is a functional amyloid that plays a key regulatory role for long-term memory in *Drosophila*. Orb2 has a glutamine, histidine-rich (Q/H-rich) domain that resembles the Q/H-rich, metal binding domain of the Hpn-like protein (Hpnl) found in *Helicobacter pylori*. In the present study, we used chromatography and isothermal titration calorimetry (ITC) to show that the Q/H-rich domain of Orb2 binds Ni^2+^ and other transition metals ions with μM affinity. Using site directed mutagenesis, we show that several histidine residues are important for binding. In particular, the H61Y mutation, which was previously shown to affect the aggregation of Orb2 in cell culture, completely inhibited metal binding of Orb2. Finally, we used thioflavin T fluorescence and electron microscopy images to show that Ni^2+^ binding induces the aggregating of Orb2 into structures that are distinct from the amyloid fibrils formed in the absence of Ni^2+^. These data suggest that transition metal binding might be important for the function of Orb2 and potentially long-term memory in *Drosophila*.

## 1. Introduction

**Figure 1:**
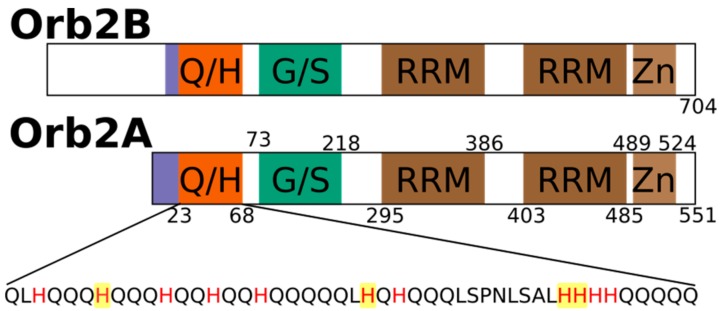
Domain structure of rare isoform Orb2A that is necessary to initiate fibril formation and the predominant isoform Orb2B highlighting the glutamine, histidine rich domain (Q/H), the glycine, serine rich region (G/S), the two RNA recognition motifs (RRM) and the zinc finger (Zn) motif. The sequence of the Q/H-rich domain is shown with all histidines in red and the hisitidines that were mutated in this study highlighted in yellow.

The aggregation of proteins into amyloid fibrils is thought to be a sporadic event in the case of amyloid diseases. However, for functional amyloids, this aggregation is expected to be a tightly regulated process. To understand how the formation of functional amyloid is regulated, we are studying factors that induce and prevent the aggregation of the functional amyloid Orb2 from *Drosophila melanogaster*. Orb2 belongs to the class of cytoplasmic polyadenylation element binding proteins (CPEBs), which are extranuclear regulators of protein expression [1]. CPEBs bind to the cytoplasmic polyadenylation elements in the 3′ untranslated region of dormant mRNAs thereby inducing their polyadenylation and translation. In *Aplysia*, the neuronal isoform of CPEB (ApCPEB), which is a key regulator of long-term potentiation (LTP), is activated by undergoing a conformational change from a soluble, oligomeric to an aggregated, amyloid-like state [2,3]. Furthermore, ApCPEB has prion-like properties when introduced into yeast and can form amyloid fibrils in vitro [4,5]. Orb2 is a CPEB homologue in *Drosophila melanogaster* which acts as a key regulator of synapse specific protein expression that is important for maintaining memories beyond 24 h [6,7,8,9,10,11]. Orb2 has two isoforms, the rare isoform Orb2A and the predominant isoform Orb2B. As shown in Figure 1, Orb2A and Orb2B share the same C-terminus, which is comprised of two RNA recognition motifs (RRMs) and a zinc finger (Zn). Both isoforms also share a common N-terminal glutamine, histidine rich (Q/H-rich) domain, which is similar to the glutamine, asparagine rich domains that are, e.g., found in yeast prions. However, Orb2A and Orb2B differ at their N-termini as Orb2A only has 21 residues preceding the Q/H-rich domain with residues 1–9 being unique to Orb2A. On the other hand, the N-terminus of Orb2B is 178 residues long.

The N-terminus of Orb2A was found to be important for amyloid formation and long-term memory, as N-terminal deletions prevented long-term memory formation [7] and even point mutations in the N-terminal domain reduced the aggregation propensity of Orb2A in vitro and long-term memory formation in flies [12,13]. We recently found that the N-terminal 21 residues preceding the Q/H-rich domain of Orb2A are sufficient for fibril formation. They form an in-register parallel β-sheet and comprise the static amyloid core of Orb2A1-88 [14]. We also showed that the same N-terminal region of Orb2A can bind to negatively charged lipid vesicles and subsequently form a helical structure that prevents the formation of amyloid fibrils [15]. Surprisingly, the Q/H-rich region of Orb2A1-88 was neither part of the amyloid core nor involved in lipid binding. This poses the following question: what is the role of the Q/H-rich domain of Orb2 if it is not involved in the formation of the amyloid core as originally thought? Interestingly, the Q/H-rich region of Orb2 strikingly resembles the glutamine and histidine rich bacterial Hpn-like protein (Hpnl) found in *Helicobacter pylori*, which colonizes the human gut. Hpnl has specific Ni^2+^, Cu^2+^, Co^2+^, and Zn^2+^ binding activity that plays a role in nickel storage, which is important for the colonization of the host [16]. Several histidine residues of Hpnl were found to be essential for metal binding, while the glutamine residues were responsible for stabilizing the Hpnl-metal complex [16,17].

The high histidine content in Orb2’s N-terminus and its similarity to Hpnl suggests that Orb2 might be able to bind transition metals similar to Hpnl. Interestingly, several other amyloid forming proteins, such as amyloid beta (Aβ), α-synuclein, and the prion protein (PrP), have metal binding activity [18]. In the case of Aβ, Cu^2+^ and Zn^2+^, binding not only modulates aggregation kinetics but was also hypothesized to cause toxicity [19].

In this paper, we ask the following questions: First, does the N-terminal Q/H-rich domain of Orb2 bind transition metals similar to HpnI and other amyloids? Second, which histidine residues are most important for metal binding? Third, what is the effect of the Orb2 metal interaction on the ability of Orb2 to form amyloid fibrils in vitro? In the following, we describe the affinity of Orb2A1-87 (Orb2A87) to several transition metal ions and characterize the effect of metal binding on aggregation kinetics. These results lead to some new hypotheses suggesting that metal interaction might also play a role in modulating the aggregation kinetics of Orb2 in vivo and could play a role in the regulation of long-term memory in *Drosophila*.

## 2. Results

### 2.1. The N-Terminus of Orb2A Binds Transition Metals in the Absence of a Polyhistidine-Tag

**Figure 2:**
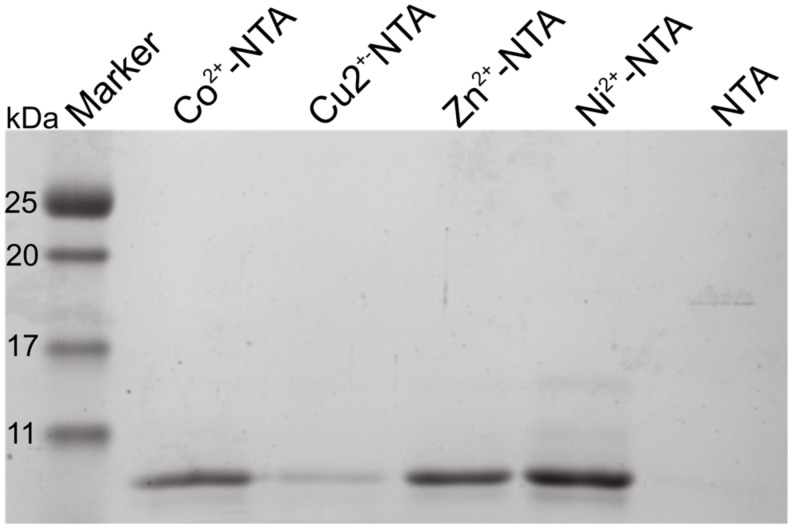
Orb2A87 binds several transition metal ions in the absence of a polyhistidine-tag. Coomassie stained SDS-PAGE showing the purification results of Orb2A87 using nitrilotriacetic acid (NTA) agarose charged with nickel, copper, cobalt, and zinc as well as metal-free NTA agarose. Orb2A87, unlike Orb2A88, has no polyhistidine-tag bound to all of these metals, but did not bind to metal-free NTA agarose.

To test whether Orb2 binds metals as hypothesized, we cloned the first 88 residues of Orb2A (Orb2A88-His) with a C-terminal polyhistidine-tag and later removed the polyhistidine-tag by mutating in an earlier stop-codon (Orb2A87). Both Orb2A88-His and Orb2A87 bind to nickel charged, nitrilotriacetic acid (Ni-NTA) resin although the binding affinity of Orb2A87 is reduced (data not shown). All experiments described in the following were done on Orb2A87, i.e., without additional polyhistidine-tag. To test whether Orb2A87 binds to metals other than nickel, we tested Orb2A87 binding to NTA resin that was stripped from metal ions and re-charged with Ni^2+^, Cu^2+^, Co^2+^, or Zn^2+^. Soluble recombinant Orb2A87 was bound to these resins at pH 8.0 and eluted from the resin at pH 3.75, at which point the histidine residues are expected to be protonated and lose their metal-binding affinity. As can be seen from Figure 2, Orb2A87 is able to bind to all of these metals ions, but not to the NTA resin without chelated bivalent metal ions. Although the same amount of protein was loaded to the same amount of resin, the intensities of the elution from the Cu-NTA resin is lower, probably because of the lower specificity and higher affinity of this resin [20].

### 2.2. Isothermal Titration Calorimetry to Characterize Metal Binding Affinity of Orb2A87

**Figure 3:**
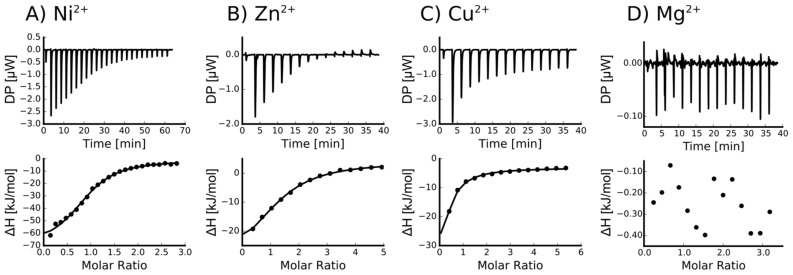
Orb2A87 binds to Ni^2+^, Zn^2+^ and Cu^2+^ but not to Mg^2+^. Isothermal titration calorimetry (ITC) titration curves of Orb2A87 with the addition of Ni^2+^ (**A**); Zn^2+^ (**B**); Cu^2+^ (**C**); and Mg^2+^ (**D**). Both the heat compensation in the sample cell containing Orb2A87 as a function of time (top) and the resulting enthalpy changes as a function of molar addition of metal and the best fit to single binding site model (bottom) are shown.

To determine the dissociation constant and number of binding sites of the metal–Orb2A87 complex, we measured isothermal titration calorimetry (ITC) using the following metals ions: Ni^2+^, Cu^2+^, Zn^2+^, Ca^2+^, and Mg^2+^. To slow amyloid formation during these measurements, we measured ITC in the presence of 1 M urea (see Materials and Methods section). Orb2A87 did not have any measurable binding affinity to Ca^2+^ and Mg^2+^. However, we measured μM dissociation constants for Ni^2+^, Cu^2+^, and Zn^2+^ (see Figure 3). The ITC data of both Ni^2+^ and Zn^2+^ fit well to a model of approximately one binding site per monomer. The results of the fits are summarized in Table 1. The binding parameters for Cu^2+^ could not be determined with confidence because it could only be measured in the presence of a weak ligand (glycine), which complicated its analysis [18]. However, the titration curve in Figure 3c indicates that Cu^2+^ has the highest affinity of all metals.

### 2.3. Several Histidines Are Important for the Metal Binding Affinity of Orb2A87

**Figure 4:**
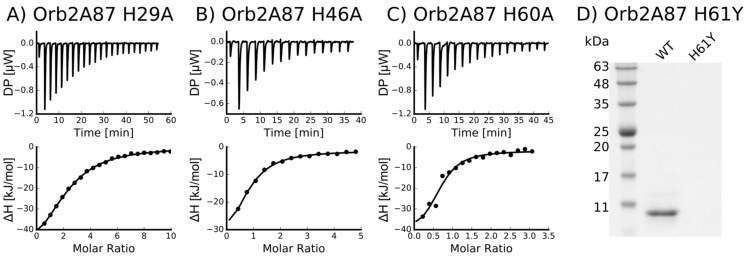
Orb2A87 mutants H29A, H46A and H60A still bind to Ni^2+^ but only with half a binding site per monomer (**A**–**C**). ITC Ni^2+^ titration curves of Orb2A87 mutants, H29A (**A**); H46A (**B**); H60A (**C**). Both the heat compensation in the sample cell containing Orb2A87 as a function of time (top) and the resulting enthalpy changes as a function of molar addition of metal and the best fit to single binding site model (bottom) are shown; (**D**) H61Y does not bind to Ni-NTA resin. SDS-PAGE comparing the Ni-NTA pH 4.25 elution fraction of wild type (WT) Orb2A87 with Orb2A87 H61Y.

To determine the location of the metal binding site of Orb2A87, we introduced several point mutations in the Q/H-rich region, H29A, H46A, H60A, and H61Y. We mutated histidine to tyrosine in the case of H61 because the H61Y mutation was previously shown by Majumdar and co-workers to limit the ability of Orb2A to aggregate in S2 cells [12]. Otherwise, we preferred the more neutral mutation from histidine to alanine. Orb2A87 H29A, H46A, and H60A could still be purified using Ni-NTA affinity chromatography indicating that the metal binding properties of these mutants remained intact. However, H61Y could not be purified this way, indicating that this mutation entirely disrupted the metal affinity of the protein (see Figure 4D). The ITC data shown in Figure 4A–C confirm that H29A, H46A, and H60A still bind nickel. As can be seen from Table 2, the nickel dissociation constants of H29A, H46A, and H60A are still in the μM range, while the number of binding sites (*n*) is decreased to less than 1 per monomer of Orb2A87.

### 2.4. Orb2A–Metal Interaction Affects the Aggregation of Orb2A87

**Figure 5:**
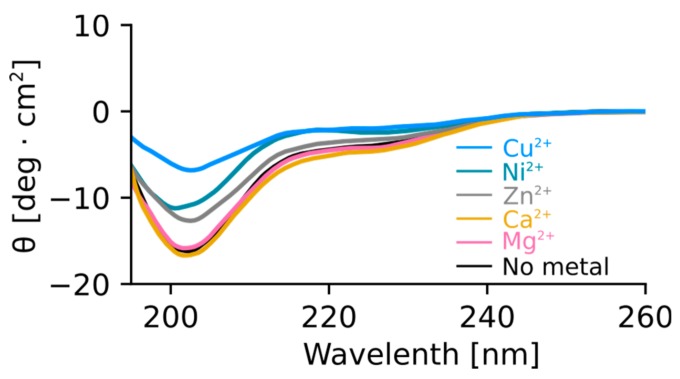
Mg^2+^ and Ca^2+^ do not change the structure of Orb2A87. Cu^2+^, Ni^2+^, and Zn^2+^ lead to a decrease in circular dichroism (CD) signal. CD spectra of Orb2A87 without metal and in the presence of 2.5 molar surplus of Cu^2+^, Ni^2+^, Zn^2+^, Ca^2+^, and Mg^2+^. The presence of Ca^2+^ and Mg^2+^ does not change the CD spectrum compatible with Orb2A87 in a predominantly random coil conformation. The addition of Cu^2+^, Ni^2+^, Zn^2+^ leads to a decay of CD signal with no indication of additional secondary structure.

To test the effect of metal binding on the structure of Orb2A87, we recorded circular dichroism (CD) spectra of Orb2A87 in the presence and absence of several metal ions. As can be seen from Figure 5, Orb2A87 has a predominantly random coil conformation in the absence of metal ions as indicated by the minimum at about 202 nm. The addition of the non-binding metals Mg^2+^ and Ca^2+^ did not have any effect on the CD spectrum of Orb2A87. Cu^2+^, Ni^2+^, and Zn^2+^, which bind to Orb2A87, led to a decrease in the CD signal. The overall shape of these less intense spectra stayed the same giving no indication of structural changes. However, the reduction of the CD signal and the absence of an isosbestic point also indicate that part of the sample aggregated and was thus not detected in the presence of Cu^2+^, Ni^2+^, and Zn^2+^.

**Figure 6:**
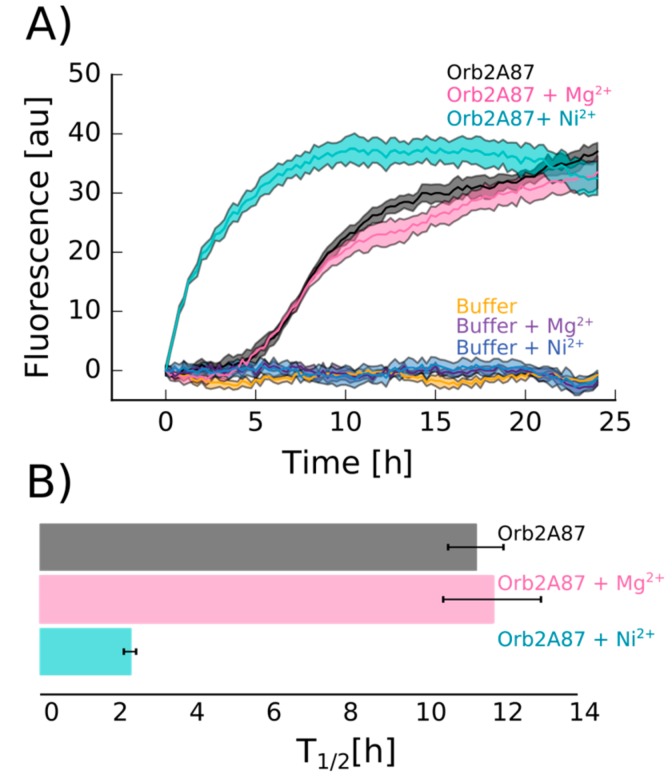
Addition of Ni^2+^ induces rapid increase of thioflavin T (Tht) fluorescence. (**A**) Tht fluorescence kinetics of 17.64 μM Orb2A87 in the absence of metal (black) and in the presence of 2.5 molar excess of Ni^2+^ (cyan) and Mg^2+^ (pink). Buffer control with and without metals shows no fluorescence increase. The average and error of three technical repeats is shown; (**B**) Average of the Tht kinetics half times (T_1/2_) calculated from three independent repeats of the Tht time courses shown in (**A**).

**Figure 7:**
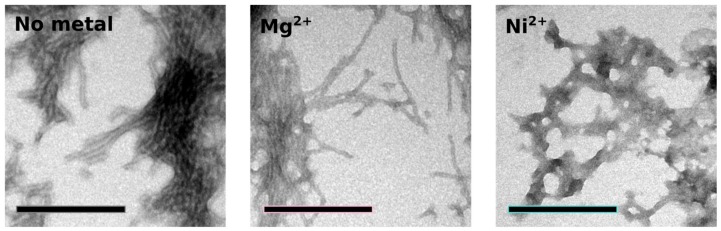
Addition of Ni^2+^ induces aggregation distinct from fibril formation. Electron micrographs of negatively stained aggregates of Orb2A87 collected 6 hours after the start of an aggregation study. Orb2A87 without metal and in the presence of a 2.5 molar excess of Mg^2+^ show typical bundled amyloid fibrils. Aggregates that formed in the presence of a 2.5 molar excess of Ni^2+^ look rather amorphous although high Tht fluorescence was observed in this case. Scale bars: 200 nm.

To study the effect of metal binding on Orb2A87 aggregation and amyloid formation, we measured thioflavin T (Tht) fluorescence kinetics in the absence of metal and in the presence of Ni^2+^. We used Mg^2+^, which did not bind to Orb2A87, as a negative control. As can be seen from Figure 6, the addition of Ni^2+^ results in an immediate increase in Tht fluorescence, whereas Orb2A87 alone or in the presence of Mg^2+^ shows an increase in fluorescence after a lag phase of about 3 h. After 10 h, the Tht fluorescence in the presence of Ni^2+^ starts to decrease again whereas it keeps increasing in the absence of Ni^2+^. To test whether the immediate increase in Tht fluorescence with the addition of Ni^2+^ was the result of metal induced fibril formation, we took electron microscopy (EM) images from samples that were negatively stained 6 h after the beginning of the aggregation study (i.e., when the Tht fluorescence of the sample containing Ni^2+^ was close to its maximum). As can be seen in Figure 7, both Orb2A87 in the absence of metal or in the presence Mg^2+^ shows the formation of bundled fibrils. However, in the presence of Ni^2+^, no clear fibrillar structures could be observed and the aggregates looked rather non-specific.

## 3. Discussion

### The N-Terminus of Orb2A Has an Affinity for Bivalent Metal Ions

In their study of Hpnl, Zeng et al. found that Hpnl binds bivalent metal ions using ITC. The presence of a similar histidine-rich domain in Orb2 led us to hypothesize that it too possesses a similar binding affinity for bivalent metal ions. This study confirms this hypothesis using metal affinity chromatography. As shown in Figure 2, Orb2A87 binds to Ni^2+^, Zn^2+^, Cu^2+^, and Co^2+^ chelated to NTA resin. The fact that Orb2A87 did not also elute from the Cu-NTA column, might be a result of its higher binding affinity to this metal [21,22]. Our ITC data in Figure 3 specify that Orb2A87 has one binding site for Ni^2+^, Zn^2+^, Cu^2^ with micromolar affinity. However, Orb2A87 does not bind Mg^2+^ or Ca^2+^ ions, indicating that the metal binding affinity of Orb2 is specific to transition metal ions. Because the Cu^2+^ binding experiment had to be performed in the presence of glycine, a weak ligand, quantitative analysis was not performed, although this interaction seems to have the lowest dissociation constant. Point mutations of several histidine residues in the Q/H-rich region showed that Orb2A87 H61Y abolishes Ni^2+^ binding. In contrast Orb2A87 H29A, H46A, and H60A, still bound Ni^2+^ with micromolar affinity although we observed an apparent decrease in the number of binding sites per Orb2A87 monomer. This reduced number of binding sites suggests that the absence of histidine residues at position 29, 46, and 60 may either reduce the number of monomers capable of binding metal, or require two protein molecules to maintain the metal binding properties of the protein. These observations confirm that the metal binding site of Orb2A87 is indeed located in the Q/H-rich region, specifically in the latter half of the domain, with the H61 residue being necessary for binding. Our CD spectra show that soluble Orb2A87 is mostly disordered in the absence of Ni^2+^, Zn^2+^, Cu^2+^, which is in agreement with our previous CD and Electron Paramagnetic Resonance (EPR) data on the soluble form of Orb2A1-88 [15]. Finally, our CD, Tht, and EM data show that Ni^2+^ binding induces the formation of Tht active Orb2A87 aggregates that are different from the amyloid fibrils observed in the absence of metal. Although an increase in Tht fluorescence has been shown to be relatively specific to amyloid fibrils [23], there are examples of similar increases in fluorescence when Tht binds to monomeric proteins [24] and amorphous aggregates [25]. Although aggregates formed in the presence of Ni^2+^ do not resemble amyloid fibrils, the increase in Tht fluorescence indicates that a Tht binding pocket and possibly a cross β-sheet rich structure is formed upon metal binding.

In summary, our data confirm the original hypothesis that the Q/H rich domain of Orb2 can bind to transition metals via its His residues. Hence, the Q/H rich domain behaves like other H-rich domains, most of which were shown to be metal binding domains. Examples of such domains not only include the Q/H-rich domains of HpnI, but also H-rich domains that are not Q-rich such as the histidine-rich glycoproteins (HRG), Cu and Zn-superoxide dismutase found in *Haemophilus*, and histidine-proline-rich glycoprotein (HPRG) [26,27,28].

What is the role of the glutamines in Orb2 in the context of metal binding? One possibility is that they help stabilize the metal binding complex similar to Hpn1 [17]. Another possibility is that metal binding induces a structural change, or stabilizes a specific conformation of the glutamines, or both. Finally, metal binding could play a role in stabilizing potential protein–protein interaction of the Q/H-rich domain. Polyglutamine (Poly-Q) domains have been suggested to be involved in coiled-coil protein–protein interfaces [29]. The additional histidines could potentially initiate and strengthen this interaction upon metal binding. For example, ligand interaction of HRGs is enhanced by its interaction with Zn^2+^ [26]. When the Q/H-rich domain of Orb2 is plotted on a helical wheel, the first six histidines fall on the same side, highlighting the potential of a metal stabilized zipper motif for Orb2. Our data clearly show that metal interaction does affect the structure of Orb2A87. However, it will be important to find out in which structural contexts metal binding occurs to determine its exact structural role.

What is the potential function of this metal interaction in long-term memory of *Drosophila*? Majumdar and co-workers showed that the H61Y mutant affected puncta formation of full length Orb2A in S2 cells. They further showed that a different mutation with a similar effect on puncta formation also inhibited long-term memory in flies [12]. Interestingly, H61Y was the only histidine mutation that completely abolished metal binding of Orb2A87. These data suggest that the metal interaction of Orb2 might play a role in its ability to aggregate in cells. Our data show that metal interaction has a pronounced effect on the structure of Orb2A87. We think that the same transition metals will also bind to the Q/H rich domain of full length Orb2A and Orb2B and have a similar effect on the structure of these proteins. Furthermore, it is possible that in a cellular environment this interaction is crucial for Orb2 aggregation to occur. Since the Q/H rich domain is part of both the rare isoform Orb2A and the predominant isoform OrbB, the effect of metals might be the same for both isoforms and not necessarily be involved in aggregation initiation which requires the unique N-terminus of Orb2A.

If transition metals are an important factor for the function of Orb2, which metal is most likely binding Orb2 in vivo? Several metals have been shown to be important for memory formation. For example, iron and zinc deficiencies affect neuronal development and can lead to memory loss [30,31,32]. In particular, Zn^2+^ has been associated with memory [33]. It has been shown that Zn^2+^ enters the presynaptic and postsynaptic neurons when released from synaptic vesicles together with glutamate and that this translocation is important for LTP at mossy fibers in rat [34]. Zinc could similarly act as a second messenger on Orb2 during long-term memory formation in *Drosophila* by introducing a structural change or by regulating protein–protein interactions. The basal concentration of free Zn^2+^ in the cytosol is <10^−9^ M, well below the dissociation constant that we determined via ITC. However, during excitation, the intracellular Zn^2+^ concentration can significantly increase [35] potentially inducing structural changes in the Q/H-rich domain of Orb2. The role of these structural changes could be either to initiate the formation of functional Orb2 aggregates, or to induce a non-amyloidogenic, reversible off pathway aggregation that would postpone amyloid formation until the Zn^2+^ concentration goes back to equilibrium.

Further research is necessary to reveal the role of metal binding on the glutamine/histidine rich domain of Orb2 and the effect of this interaction on long-term memory in *Drosophila*.

## 4. Materials and Methods

### 4.1. Cloning of Orb2A Mutants

The original pET DEST42 plasmid for Orb2A expression in *Escherichia coli* was provided by Dr. Kausik Si. Starting from this plasmid, we cloned the N-terminal 88 residues of Orb2A into a pET28b vector at the 5′ NcoI and 3′ XhoI restriction sites. The resulting Orb2A88 Y2G mutation was changed back to the wild type via QuickChange site-directed mutagenesis (Agilent, Santa Clara, CA, USA). The following other mutations were made using QuickChange site-directed mutagenesis: Orb2A87 without polyhistidine-tag (additional stop codon); Orb2A87 H29A; H46A; H60A; H61Y.

The success of cloning and mutagenesis was confirmed via plasmid DNA sequencing.

Primers used (all written from 5′ to 3′):

Orb2A88Y2G NcoI GCCCCCTTCACCATGGGCAACAAATTTG
Orb2A88Y2G XhoI ATAAGCTCGAGCGATCCTCCGCCTCCTCCACC
Orb2A88WTsense GAAGGAGATATACCATGTACAACAAATTTGTTAATTTCATTTGCGGTG
Orb2A88 WT antisense ACCGCAAATGAAATTAACAAATTTGTTGTACATGGTATATCTCCTTC
Orb2A87 No polyhistidine-tag sense GAGGAGGCGGAGGATAGCTCGAGCACC
Orb2A87 No polyhistidine-tag antisense GGTGCTCGAGCTATCCTCCGCCTCCTC
Orb2A87 H29A sense AGCTCCACCAGCAACAGGCTCAACAACAGCATCAGC
Orb2A87 H29A antisense GCTGATGCTGTTGTTGAGCCTGTTGCTGGTGGAGCT
Orb2A87 H46A sense AACAGCAGCAACAGCTCGCTCAGCACCAACAGCAAC
Orb2A87 H46A antisense GTTGCTGTTGGTGCTGAGCGAGCTGTTGCTGCTGTT
Orb2A87 H60A sense GAATCTGAGTGCCCTGGCCCATCATCACCAGCAG
Orb2A87 H60A antisense CTGCTGGTGATGATGGGCCAGGGCACTCAGATTC
Orb2A87 H61Y sense CTGAGTGCCCTGCACTATCATCACCAGCAGC
Orb2A87 H61Y antisense GCTGCTGGTGATGATAGTGCAGGGCACTCAG

### 4.2. Protein Expression

*E. coli* Rosetta 2 (DE3) (Novagen-EMD Millipore, Billerica, MA, USA) was transformed with corresponding pET28b vector. Transformed cells were grown in Luria Bertani (LB) Miller medium with 35 μg/L chloramphenicol and 50 μg/L kanamycin at 30 °C for 15–18 h. Cultures were diluted into LB Miller medium with 35 μg/L chloramphenicol and 50 μg/L kanamycin and grown at 37 °C until the optical cell density at 600 nm reached 0.6. An amount of 1 mM isopropyl 1-thiol-d-galactopyranoside (IPTG) was then added to induce protein expression. Induction proceeded for 16 h while cells remained shaking at 37 °C. Cells were pelleted by centrifugation at 4000× rpm for 20 min using a Sorvall SLC-6000 rotor (Thermo Fisher Scientific Inc., Waltham, MA, USA). Cell pellets were immediately stored at −80 °C.

### 4.3. Protein Purification

For ITC samples, cells were defrosted on ice and resuspended in denaturing buffer (8 M urea, 10 mM citrate, 100 mM sodium phosphate, 10% glycerol, 0.05% 2-mercaptoethanol) at pH 8.0. The suspended cells were further lysed by sonication for 6 minutes using a cell disruptor sonicator (Heat Systems Model W-220F Qsonica, Newtown, CT, USA). The lysate was later centrifuged at 20,000× rpm for 20 min using a Sorvall ss-34 rotor (Thermo Fisher Scientific Inc.). The resulting supernatant was loaded onto pre-equilibrated Sigma HIS-select Ni-NTA resin (Sigma-Aldrich, St. Louis, MO, USA) and incubated on a shaker at room temperature for at least 1 h. Following incubation, the flow through was collected and the column was washed with denaturing buffer at pH 8.0 to which 0.5% Triton X-100 and 500 mM NaCl were added. The column was subsequently washed with denaturing buffer pH 6.75. Protein was eluted with a pH step gradient in denaturing buffer. The protein predominantly eluted at pH 4.25. The resulting pure protein was either used immediately or frozen in 2 mL aliquots using liquid N_2_ and stored at −80 °C.

For samples used in ThT fluorescence and CD assays, protein was purified in the same manner as above. Except for the following modifications: (1) denaturing buffer contained no glycerol and 10 mM Tris instead of citrate; (2) in addition to the Triton X-100, 500 mM NaCl, and pH 6.75 washes, the column was further washed with renaturing buffer (50 mM sodium phosphate, 200 mM NaCl, 10% glycerol, 0.05% 2-mercaptoethanol at pH 8.00) and renaturing buffer containing 20 mM imidazole; (3) the protein was eluted in renaturing buffer containing 250 mM imidazole.

### 4.4. Affinity Chromatography

To examine Orb2A87 metal affinity, agarose Ni-NTA, Cu-NTA, Co-NTA or Zn-NTA resins were tested individually. Each resin was prepared by regenerating HIS-Select Ni-NTA resin with the desired metal following removal of chelated Ni from the resin according to the protocol listed in the GenScript Technical Manual No. 0237 (Genescript, Piscataway, NJ, USA). HIS-Select Ni-NTA resin was washed with 2 column volumes 6 N GuHCl, 0.2 M Acetic Acid, followed by 5 column volumes of deionized water and 3 column volumes of 2% sodium dodecyl sulfate (SDS). After a wash with 5 column volumes of deionized water, the column was washed with 5 column volumes of 100% ethanol after which the column was washed again with 5 column volumes of deionized water. To remove chelated nickel ions, the column was washed with 5 column volumes of 100 mM ethylenediaminetetraacetic acid (EDTA) pH 8.0 and 5 column volumes of deionized water. To agarose NTA beads, 5 column volumes of a 100 mM stock solution of the desired metal were added (100 mM Co(NO_3_)_2_·6H_2_O, 100 mM CuCl_2_·2H_2_O, or 100 mM ZnCl_2_). 

Following equilibration of each column with denaturing buffer, pure Orb2A87 WT was incubated on each metal affinity resin for 1 h. Following incubation, the resin was washed with denaturing buffer pH 8.0. Protein was eluted with denaturing buffer at pH 4.25 to 3.75. Protein elutions were collected and stored at 4 °C, and subsequently analyzed using SDS-PAGE and Coomassie Blue staining.

### 4.5. ITC Measurements

ITC experiments were performed at 30 °C using a MicroCal PEAQ-ITC microcalorimeter (Malvern Instruments Ltd., Malvern, UK). Orb2A87 samples were prepared using a GE Healthcare PD-10 desalting column (GE Healthcare, Buckinghamshire, UK). Sample was eluted with 1 M urea, 10 mM 4-(2-hydroxyethyl)piperazine-1-ethanesulfonic acid (HEPES), 100 mM NaCl pH 7.4, which was filtered and degassed. Protein concentration was determined using ultraviolet (UV) absorbance at 280 nm and was in the range of 20–50 μM. Metal titrants (0.5–1 mM) were prepared using the same buffer. Fifteen to 25 injections of 2.5 µL metal titrant were titrated into the sample cell over 2.5 s with a stirring speed of 750× rpm to ensure equilibration. Data were baseline adjusted by subtracting background data obtained from equivalent injections of metal titrant into the buffer solution. The titration curves were analyzed using MicroCal PEAQ-ITC analytics software (version 1.0.0.1259, Malvern Instruments Ltd.) using a model that assumes a set of identical binding sites where the total heat of the solution is calculated as:

(1) $$Q=\frac{nM_{t}\Delta HV_{0}}{2}\left(1+\frac{X_{t}}{nM_{t}}+\frac{1}{nKM_{t}}-\sqrt{{\left(1+\frac{X_{t}}{nM_{t}}+\frac{1}{nKM_{t}}\right)}^{2}-\frac{4X_{t}}{nM_{t}}}\right)$$

where *n* is the number of binding sites per monomer, *M_t_* is the total concentration of protein, Δ*H* is the molar heat of ligand binding, *X_t_* the total concentration of ligand, and *K* the binding constant $(K=1/K_{D}).$ The heat per injection *i* can then be calculated via the following equation:

(2) $$\Delta Q\left(i\right)=Q\left(i\right)+\frac{\Delta V_{i}}{V_{0}}\frac{Q\left(i\right)+Q\left(i-1\right)}{2}-Q\left(i-1\right)$$

which compensates for the additional volume $\left(\Delta V_{i}\right)$ of each injection. The parameters, *n*, *K*, and Δ*H* are determined using non-linear least square fitting using the Levenberg–Marquardt algorithm.

### 4.6. Circular Dichroism 

Purified Orb2A87 aliquots were buffer exchanged into 75 mM phosphate and 100 mM NaF buffer pH 7.6 using a PD-10 desalting column (GE Healthcare, Chicago, IL, USA). Protein concentration was determined using UV absorbance at 280 nm and calculated to be 19 μM. NiCl_2_, MgCl_2_, ZnCl_2_, CaCl_2_, and CuCl_2_ were dissolved in deionized H_2_O to make 10 mM stock solutions. Metal stocks were added to protein in a 2.5 molar excess of metal over protein, 0.98 μL of each metal stock. For protein with no metals, 0.98 μL deionized H_2_O was added. Circular dichroism was measured from 260 nm to 195 nm on a Jasco J-810 spectropolarimeter (Jasco Inc., Easton, MD, USA) at a scan speed of 50 nm/min with data points at every 0.5 nm. Background measurements were taken with buffer and metal or buffer and water to correlate with each sample. Sample and background measurements were averaged over 16 scans. The background spectra were subtracted from each respective sample spectrum and the results were plotted.

### 4.7. Thioflavin T Fluorescence Assay

The role of metal binding on amyloid formation was tested using the following thioflavin T binding assay. Orb2A87 samples were prepared using GE Healthcare PD-10 desalting columns, and a 20 mM HEPES, 100 mM NaCl pH 7.4 buffer. The eluted samples were pooled and then aliquoted into three fractions of equal volume. Each fraction was aggregated in the presence or absence of the appropriate ligand (Ni^2+^ or Mg^2+^) in a 2.5:1 ligand to protein molar ratio. Samples were measured in triplicate (200 μL fractions plus 2.5 μL of 5 mM thioflavin T stock), using an Eppendorf Plate Reader AF2200 fluorimeter (Eppendorf, Hauppauge, NY, USA). Samples were excited at 440 nm with bandwidth of 20 nm and emission was recorded at 484 nm with a bandwidth of 25 nm. Fluorescence measurements were taken every 15 min, with 2 s shaking prior to measurement, and over the course of 24 h. Collected data points were averaged and the standard deviation was calculated.

### 4.8. Electron Microscopy

To observe the morphology of aggregate species used in the thioflavin T binding assay, electron micrographs were prepared for each of the samples described above. A 10 μL drop of the sample was placed on a sheet of parafilm and a copper mesh electron microscopy grid (Electron Microscopy Sciences, Hatfield, PA, USA) was incubated with the sample for 2 min. Following incubation and removal of excess liquid, 10 μL of 1% uranyl acetate solution was placed on the grid and incubated for 30 s. Upon removal of excess liquid, the grid was washed with 10 μL of 1% uranyl acetate and dH_2_O, and was dried for 1 h. Grids were imaged using a JEOL JEM-1400 electron microscope (JEOL, Peabody, MA, USA) at 100 kV and photographed using a Gatan digital camera (Gatan Inc., Pleasanton, CA, USA).

## Figures and Tables

**Table 1:** Thermodynamic values for Orb2A87 binding to divalent metal ions as derived from fitting the ITC data shown in Figure 3 to a model assuming a single binding site.

Ligand	*n* (sites)	*K*_D_ (M)	Δ*H* (kJ/mol)	Δ*G* (kJ/mol)	−*T*Δ*S* (kJ/mol)
Ni^2+^	0.915 +/− 2.2·10^−2^	4.66e^−6^ +/− 758·10^−9^	−69.4 +/− 3.57	−31.0	38.4
Zn^2+^	1.29 +/− 5.1·10^−2^	12.6e^−6^ +/− 2.13·10^−6^	−37.8 +/− 3.53	−28.5	9.38
Cu^2+^	Not determined *	-	-	-	-
Ca^2+^	Not binding	-	-	-	-
Mg^2+^	Not binding	-	-	-	-

* For Cu^2+^, the binding parameters were not determined since Cu^2+^ binding could only be measured in the presence of a weak binder (Glycine). The number equivalent binding sites is *n*, *K*_D_ is the dissociation constant, Δ*H* the molar heat, Δ*G* the free energy, and Δ*S* the entropy of ligand binding.

**Table 2:** Thermodynamic values for Orb2A87 mutants binding to Ni^2+^ derived from fitting the ITC data shown in Figure 4.

Mutant	*n* (sites)	*K*_D_ (M)	Δ*H* (kJ/mol)	Δ*G* (kJ/mol)	−*T*Δ*S* (kJ/mol)
H29A	0.412 +/− 1.1·10^−2^	8.21·10^−6^ +/− 805·10^−9^	−61.3 +/− 3.48	−29.5	31.7
H46A	0.715 +/− 6.8·10^−2^	4.14·10^−6^ +/− 809·10^−9^	−41.9 +/− 6.12	−31.3	10.7
H60A	0.652 +/− 6.8·10^−2^	2.49·10^−6^ +/− 1.33·10^−6^	−41.9 +/− 8.06	−32.5	9.41
H61Y	Not binding *	-	-	-	-

* H61Y could not be purified via a Ni-NTA column.
